# Prognostic value of plasma microRNAs for non-small cell lung cancer based on data mining models

**DOI:** 10.1186/s12885-024-11830-9

**Published:** 2024-01-10

**Authors:** Mengqing Yan, Wenjun Kang, Xiaohua Liu, Bin Yang, Na Sun, Yongli Yang, Wei Wang

**Affiliations:** 1 Department of Occupational and Environmental Health, College of Public Health, Zhengzhou University, Zhengzhou, China; 2 The Key Laboratory of Nanomedicine and Health Inspection of Zhengzhou, Zhengzhou University, Zhengzhou, China; 3 Zhuji People’s Hospital of Zhejiang Province, Shaoxing, China; 4 Department of Epidemiology and Biostatistics, College of Public Health, Zhengzhou University, Zhengzhou, China

**Keywords:** Non-small cell lung cancer, MicroRNA, Prognosis, Data mining, Prediction

## Abstract

**Background:**

As biomarkers, microRNAs (miRNAs) are closely associated with the occurrence, progression, and prognosis of non-small cell lung cancer (NSCLC). However, the prognostic predictive value of miRNAs in NSCLC has rarely been explored. In this study, the value in prognosis prediction of NSCLC was mined based on data mining models using clinical data and plasma miRNAs biomarkers.

**Methods:**

A total of 69 patients were included in this prospective cohort study. After informed consent, they filled out questionnaires and had their peripheral blood collected. The expressions of plasma miRNAs were examined by quantitative polymerase chain reaction (qPCR). The Whitney U test was used to analyze non-normally distributed data. Kaplan-Meier was used to plot the survival curve, the log-rank test was used to compare with the overall survival curve, and the Cox proportional hazards model was used to screen the factors related to the prognosis of lung cancer. Data mining techniques were utilized to predict the prognostic status of patients.

**Results:**

We identified that smoking (*HR* = 2.406, 95% *CI* = 1.256–4.611), clinical stage III + IV (*HR* = 5.389, 95% *CI* = 2.290-12.684), the high expression group of miR-20a (HR = 4.420, 95% CI = 1.760–11.100), the high expression group of miR-197 (HR = 3.828, 95% CI = 1.778–8.245), the low expression group of miR-145 ( HR = 0.286, 95% CI = 0.116–0.709), and the low expression group of miR-30a (HR = 0.307, 95% CI = 0.133–0.706) was associated with worse prognosis. Among the five data mining models, the decision trees (DT) C5.0 model performs the best, with accuracy and Area Under Curve (AUC) of 93.75% and 0.929 (0.685, 0.997), respectively.

**Conclusion:**

The results showed that the high expression level of miR-20a and miR-197, the low expression level of miR-145 and miR-30a were strongly associated with poorer prognosis in NSCLC patients, and the DT C5.0 model may serve as a novel, accurate, method for predicting prognosis of NSCLC.

**Supplementary Information:**

The online version contains supplementary material available at 10.1186/s12885-024-11830-9.

## Introduction

Lung cancer is the second most commonly diagnosed cancer and the leading cause of cancer death, representing approximately 11.4% cancers diagnosed and 18.0% deaths, with an estimated 2.2 million new cancer cases and 1.8 million deaths worldwide in 2020 [1]. Lung cancer can be divided into small cell lung cancer (SCLC) and non-small cell lung cancer (NSCLC) according to pathological types, with NSCLC accounting for more than 80% of all lung cancers [2]. NSCLC can be further divided into adenocarcinoma (AC), squamous cell carcinoma (SCC) and large cell carcinoma (LCLC) three major histological subtypes. The NSCLC patients have no obvious clinical symptoms at an early stage, and more than 75% of NSCLC patients are diagnosed in the terminal. Compared with other malignancies, lung cancer patients have a lower survival rate, with a five-year survival rate of less than 20% [3]. Previous studies have shown that the baseline patient factors after diagnosis is strongly associated with prognostic overall survival in NSCLC [4]. In addition, baseline patient factors also influence clinical treatment modalities, which in turn affects prognostic status. Therefore, it is necessary to explore the baseline and prognostic survival status of patients after diagnosis.

MiRNAs are a class of small (about 20-22nt) noncoding RNAs originally transcribed by RNA polymerase II [5]. MicroRNAs (miRNAs) play an important role in gene expression and regulation by base-complementary pairing with messenger RNAs (mRNAs) [6]. Because of its easy accessibility through blood and less invasive to patients compared to other clinical tumor markers, miRNAs are more suitable for clinical applications in tumor prognosis. In recent years, miRNAs have been found to be involved in many key biological processes, and are closely associated with cancer development as well as progression. Disease regulated miRNAs have been extensively studied in the past few years, and several blood-based miRNA tests have been developed for lung cancer diagnosis, with reasonable sensitivity and specificity [7]. For example, Rosenfeld performed miRNA analysis on 22 of the most common solid tumors and developed a 48-miRNA classifier to determine the origin of unknown primary cancers with a sensitivity of 81% [8]. MiRNA dysregulation can be detected at any stage. Sensing, from initiation to progression, allows us to observe dynamic changes in real time [9]. These findings bring hope for minimally invasive and early lung cancer diagnosis by exploiting cell-free miRNA expression behavior (cfmiRNA). A large analytical study on miRNA-based lung cancer diagnosis by Liao et al. found that a comprehensive biomarker panel composed of plasma miRNA and sputum miRNA significantly improved the sensitivity and specificity of lung cancer diagnosis [10]. Our research found that the expression levels of miR-21, miR-20a, miR-210, miR-145, miR-126, miR-223, miR-197, miR-30a, miR-30d, and miR-25 in plasma of lung cancer patients were correlated with diagnosis of lung cancer [11], but the role of these miRNAs in the prognosis of non-small cell lung cancer has not been explored.

Data mining (DM) is extracting potentially useful information and knowledge of the process from abundant, incomplete, noisy, fuzzy and random practical application data [12]. Limited by the complexity of clinical information, traditional statistical methods are difficult to perform accurate analysis, while data mining techniques are not affected by the type of variables and the existence of non-linear relationships, and can be better applied to the analysis of clinical information. Typical machine learning methods include bayesian neural networks (BNN), artificial neural networks (ANN), linear discriminant analysis (LDA), decision trees (DT), and support vector machines (SVM). Nowadays, data mining of biomarkers has been extensively used for mechanistic studies, diagnosis and prognosis of diseases, which appears to be particularly common in the field of cancer [13, 14].

Among the available data mining studies on biomarkers of lung cancer, most of them have focused on studies of public databases, such as Liu et al. used the Gene Expression Omnibus (GEO) data sets to screen potential immune-related genes and used data mining techniques to draw the conclusion that gene OLR1 played a key role in tumor immune microenvironment and could predict or potentially be regulated for NSCLC immunotherapy [15]. However, data mining studies of peripheral blood miRNA-based prognostic biomarkers for NSCLC in clinical participants have been seldom reported. In this work, based on the clinical information and the expression of plasma miRNAs, we screened the risk factors for disease prognosis and established the best prognostic model to provide a guideline for NSCLC prognosis as well as treatment.

## Results

### Relationship between demographic characteristics and prognosis in NSCLC patients

As of August 2021, 69 NSCLC patients (mean age was 60.61 ± 8.63 years, median survival time was 30.00 (14.50, 56.00) months) were enrolled. An unadjusted test showed that clinical stage was statistically associated with the prognosis of NSCLC patients (*P* < 0.15); Age, gender, smoking, and alcohol consumption were not statistically significant with the prognosis of NSCLC patients (*P* > 0.15) (Table 1).

**Table 1:** Demographic characteristics of NSCLC patients

Variables	Cases (*n* = 69)	Overall Survival (months)^a^	χ^*2*^	*P*
Age (years)				
≤ 60	31	35.00 (16.00, 56.50)	1.299	0.254
> 60	38	22.50 (10.50, 55.00)		
Gender				
Male	44	24.00 (15.00, 54.50)	1.031	0.310
Female	25	38.00 (14.00, 56.00)		
Smoking				
Yes	33	25.00 (8.00, 54.00)	1.112	0.292
No	36	32.00 (15.75, 56.00)		
Drinking				
Yes	5	17.00 (6.00, 57.00)	0.063	0.803
No	64	31.00 (15.00, 56.00)		
Pathological type				
Squamous carcinoma	24	30.50 (10.25, 54.50)	1.151	0.562
Adenocarcinoma	44	29.00 (15.75, 56.00)		
Large cell carcinoma	1	-		
Clinical Stage				
I + II	24	55.00 (43.75, 58.25)	14.968	< 0.001
III + IV	45	20.00 (10.00, 38.00)		

^a^Overall survival was expressed by T_50_ (T_75_, T_25_)

### The relationship between miRNA expression and prognosis for NSCLC patients

**Fig. 1:**
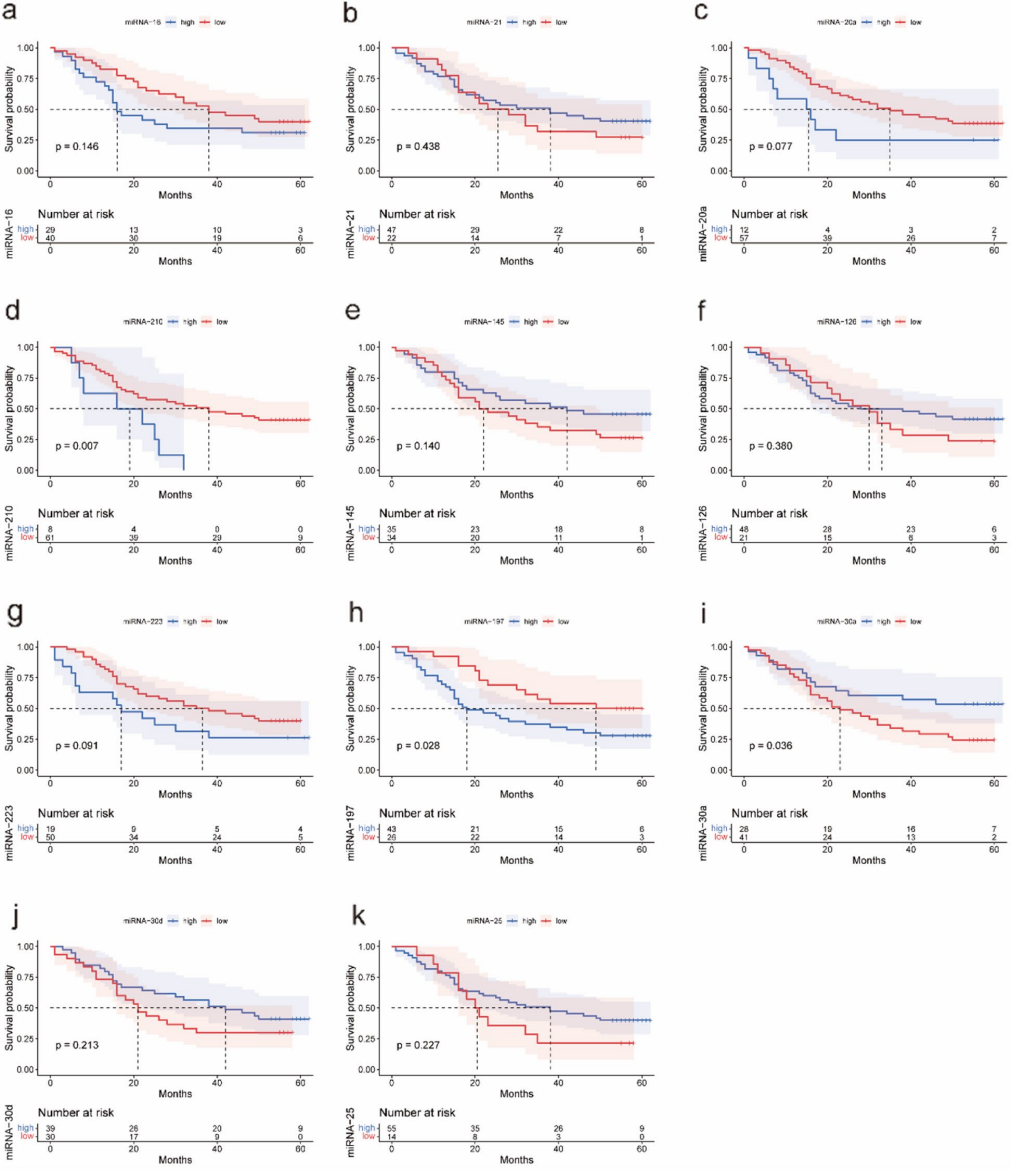
Survival curves of miRNAs expression levels. **a** Survival curves of miRNA-16; **b** Survival curves of miRNA-21; **c** Survival curves of miRNA-20a; **d** Survival curves of miRNA-210; **e** Survival curves of miRNA-145; **f** Survival curves of miRNA-126; **g** Survival curves of miRNA-223; **h** Survival curves of miRNA-197; **i** Survival curves of miRNA-30a; **j** Survival curve of miRNA-30d; **k** Survival curve of miRNA-25

Based on the survival status and expression of miRNAs, receiver operating characteristic (ROC) curves were used to determine the cutoff value for the expression of 11 miRNAs in NSCLC patients. The cut-off values for miRNA-16, miRNA-21, miRNA-20a, miRNA-210, miRNA-145, miRNA-126, miRNA-223, miRNA-197, miRNA-30a, miRNA-30d and miRNA-25 were 1.79, 0.83, 7.18, 4.09, 1.08, 0.94, 4.04, 0.78, 1.03, 1.12, and 0.58, respectively. According to the cut-off value of each miRNA, NSCLC patients were divided into high and low-expression groups, and the results are shown in Table 2. The survival curves of the 11 miRNAs are shown in Fig. 1.

**Table 2:** Log-rank test for the expression levels of 11 miRNAs in peripheral blood

Variables	Cut-off values	expression groups	Cases	Overall Survival (months)^a^	χ^*2*^	*P*
miRNA-16	1.79	Low expression	40	38.00 (18.50, 57.00)	2.111	0.146
		High expression	29	16.00 (9.50, 53.50)		
miRNA-21	0.83	Low expression	22	25.50 (15.25, 55.25)	0.602	0.438
		High expression	47	38.00 (14.00, 58.00)		
miRNA-20a	7.18	Low expression	57	35.00 (15.50, 56.00)	3.135	0.077
		High expression	12	15.50 (6.25, 46.75)		
miRNA-210	4.09	Low expression	61	38.00 (15.00, 56.50)	7.224	0.007
		High expression	8	19.00 (7.25, 25.75)		
miRNA-145	1.08	Low expression	34	22.00 (12.75, 55.25)	2.180	0.140
		High expression	35	42.00 (15.00, 59.00)		
miRNA-126	0.94	Low expression	21	30.00 (15.50, 53.00)	0.771	0.380
		High expression	48	33.00(13.25,56.00)		
miRNA-223	4.04	Low expression	50	36.50 (15.75,56.00)	2.864	0.091
		High expression	19	17.00 (6.00,57.00)		
miRNA-197	0.78	Low expression	26	51.50 (21.00,57.00)	4.853	0.028
		High expression	43	18.00 (11.00,53.00)		
miRNA-30a	1.03	Low expression	41	23.00 (12.50,52.00)	4.414	0.036
		High expression	28	53.00 (15.25,59.75)		
miRNA-30d	1.12	Low expression	30	21.00 (11.00,56.00)	1.548	0.213
		High expression	39	42.00 (15.00,59.00)		
miRNA-25	0.58	Low expression	14	20.50 (14.75,40.00)	1.461	0.227
		High expression	55	38.00 (14.00,56.00)		

^a^Overall survival was expressed by T_50_ (T_75_, T_25_)

### Multivariate analysis of prognosis of NSCLC

To improve the power of testing for the Cox proportional hazards regression model, the significance level α for the one-way analysis was widened to 0.15, and the variables included were clinical stage, miRNA-16, miRNA-20a, miRNA-210, miRNA-145, miRNA-223, miRNA-197, and miRNA-30a; in addition, age and smoking were included in the model in conjunction with clinical common sense finally. The results showed in Table 3 that smoking (*HR* = 2.406, 95% *CI*: 1.256–4.611), clinical stage III + IV (*HR* = 5.389, 95% *CI*: 2.290–12.684), miRNA-20a high expression group (*HR* = 4.420, 95% *CI*: 1.760–11.100), miRNA-197 high expression group (*HR* = 3.828, 95% *CI*: 1.778–8.245), miRNA-145 low expression group (*HR* = 0.286, 95% *CI*: 0.116–0.709), and miRNA-30a low expression group (*HR* = 0.307, 95% *CI*: 0.133–0.706) were associated with poorer prognosis (*P* < 0.05).

**Table 3:** The Cox proportional hazards regression model of NSCLC patients

**Variables**	*β*	*SE*	*Wald*	*P*	*HR*	95%*CI*	
						Lower	Upper
Smoking	0.878	0.332	7.004	0.008	2.406	1.256	4.611
Clinical Stage	1.684	0.437	14.878	< 0.001	5.389	2.290	12.684
miRNA-20a	1.486	0.470	10.003	0.002	4.420	1.760	11.100
miRNA-145	-1.251	0.463	7.305	0.007	0.286	0.116	0.709
miRNA-197	1.342	0.391	11.765	0.001	3.828	1.778	8.245
miRNA-30a	-1.182	0.425	7.733	0.005	0.307	0.133	0.706

The stepwise backward method was used to exclude the non-significant variables

### Data mining

#### Model predictions

Based on results of the Cox proportional hazards regression model, the variables incorporated into the data mining model were smoking, clinical stage, miRNA-20a, miRNA-145, miRNA-197, miRNA-30a. Table 4 showed that three years of survival for the prediction accuracy of the BNN model in the training set was 75.47% and 62.50% in the prediction set; the prediction accuracy of the ANN model in the training set was 86.79% and 81.25% in the prediction set; the prediction accuracy of the LDA model in the training set was 81.13% and 75.00% in the prediction set; the DT C5.0 model in the training set prediction accuracy was 94.34% and 93.75% in the prediction set; SVM model prediction accuracy was 90.57% in the training set and 87.50% in the prediction set.

**Table 4:** The results of data mining models prediction

Models	Training set (*n* = 53)		Total	Prediction set (*n* = 16)		Total
	**Survival**	**Death**		**Survival**	**Death**	
**BNN**						
Survival	15	9	24	3	4	7
Death	4	25	29	2	7	9
Total	19	34	53	5	11	16
**ANN**						
Survival	18	6	24	6	1	7
Death	1	28	29	2	7	9
Total	19	34	53	8	8	16
**LDA**						
Survival	19	5	24	5	2	7
Death	5	24	29	2	7	9
Total	24	29	53	7	9	16
**C5.0**						
Survival	21	3	24	6	1	7
Death	0	29	29	0	9	9
Total	21	32	53	6	10	16
**SVM**						
Survival	20	4	24	7	0	7
Death	1	28	29	2	7	9
Total	21	32	53	9	7	16

### Model comparison

The AUC of BNN model is less than 0.7; the AUC of LDA model and ANN model is greater than 0.7; the AUC of SVM model is close to 0.9; the AUC of DT C5.0 model is greater than 0.9, especially the sensitivity and negative prediction value of the model are 100%, and the specificity and positive prediction value are more than 80%. These results are shown in Table 5.

**Table 5:** Evaluation of data mining models prediction

Models	Accuracy (%)	Sensitivity(%)	Specificity(%)	Positive Predictive Value (%)	Negative predictive value (%)	AUC (95%*CI*)
BNN	62.50	60.00	63.64	42.86	77.78	0.603 (0.335, 0.883)
ANN	81.25	75.00	87.43	85.71	77.78	0.817 (0.549, 0.962)
LDA	75.00	71.43	77.78	71.43	77.78	0.746 (0.472, 0.925)
SVM	87.50	77.78	100.00	100.00	77.78	0.889 (0.634, 0.989)
DT C5.0	93.75	100.00	90.00	85.71	100.00	0.929 (0.685, 0.997)

## Discussion

The prognostic survival time of non-small cell lung cancer is strongly related to patient's underlying characteristics as well as biomarkers are strongly associated. In this study, smoking, clinical stage, miRNA-20a, miRNA-145, miRNA-197, and miRNA-30a were screened as prognostic risk factors in NSCLC patients. In the prediction results of the data mining model, the AUC of the DT C5.0 model was 0.929; similarly, the SVM model was 0.889; the ANN model was 0.817; the LDA model was 0.746; BNN model was 0.603.

The hazard ratio (HR)for smoking behavior and clinical stage in the multifactorial model were 2.406 and 5.389, respectively. Smoking is the most common risk factor for lung cancer, and studies have shown a clear dose–response relationship between smoking and lung cancer[16]. An estimated 75.04% of lung cancer deaths in males and 18.35% of lung cancer deaths in females in China are attributable to tobacco use [17]. Clinical stage at diagnosis is closely associated with lung cancer prognosis. Patients with stage I lung cancer have a 5-year survival rate of 60% postoperatively, while patients with stage IV lung cancer have a 5-year survival rate of < 5%. The survival rates of lung cancer patients are significantly improved by early diagnosis and timely, appropriate treatment [18]. The identification of alterations in the gene levels associated with certain types of tumors in the tissues or body fluids of patients during early tumor formation may significantly improve early diagnosis of lung cancer.

MiRNAs have received widespread attention as important regulators of the cancer genome. Many studies have shown that miRNA-20a is overexpressed in many malignancies and promotes tumor cell proliferation, migration and invasion through multiple pathways. Du et al. found that up-regulated miR-20a can activate downstream molecules such as *livin* and *survivin* through the *NF-κB* pathway, and up-regulated miR-20a can also promote the development of colorectal cancer by inhibiting various tumor suppressor genes such as *BIM* and *Smad4* [19]. Xu et al. found that miR-20a was up-regulated in the plasma of NSCLC patients, and its high expression was associated with poor prognosis [20]. The results of this study showed that high miRNA-20a expression in NSCLC patients was associated with a poorer prognosis, with a hazard ratio of 4.420 compared to the low expression group. In addition, previous studies have shown that miRNA-145 can inhibit cell proliferation by targeting the oncogene *c-Myc*, which in turn inhibits tumor cell growth [21]. Campayo et al. found that low expression of miR-145 in NSCLC tissues was associated with poor patient prognosis [22]. In this study, we found that miR-145 low expression was associated with a poorer prognosis in NSCLC patients, with a HR of 0.286 compared to the low expression group. Moreover, miRNA-197 exerts pro-cancer effects by inhibiting apoptosis of *P53* gene, including inhibition of *NOXA* and *BMF* genes. Mavridis et al. found that high expression of miR-197 was closely related to poor prognosis in NSCLC patients [23]. In this study, we found that high miR-197 expression was a risk factor for prognosis in NSCLC patients, with a risk ratio of 3.828 compared to the low expression group. Finally, miR-30a regulates many important signaling pathways such as *P53*, *PI3K*/*AKT*. It was shown that miRNA-30a can inhibit hepatocellular carcinoma cell proliferation by targeting the *MTDH/PTEN/Akt* pathway [24]. MiRNA-30a is also closely related to apoptosis in cancer cells, promoting apoptosis through the expression of down-regulated BCL-2 expression. In addition, miRNA-30a plays an important role in tumor invasion and migration [25]. In this study, we found that the miR-30a low expression group was correlated with a poorer prognosis, with a risk ratio of 0.307 compared to the low expression group.

Comparing the prediction results of the data mining models, we found a large difference in the performance. Firstly, the BNN model has the advantage of being able to work with data containing missing and disordered items. However, the BNN model is computationally slow compared to other algorithms, requires a strict form of input variables, and may suffer from overfitting. The poor prediction of the BNN model in this paper may be due to the large differences in variables, and the normalization process at the time of entering the model may produce more errors. Secondly, the LDA model has the advantage of dimensionality reduction, which greatly improves the classification efficiency, but the model relies on the distribution information of the input variables and requires normal transformation of the variables. Thirdly, the ANN model has the advantage of being good at stripping out the effects of variable nonlinearity and dealing with large data problems. However, ANN models are similar to BNN models in that they require normalization of variables, which may also contribute to the low prediction efficiency. Fourthly, the SVM model is a binary classification model that is widely used for its high accuracy and robustness, but the model is difficult to solve multi-classification problems. The area under the ROC curve of the ANN model in this study is 0.817. Xu et al. used differentially expressed genes and protein–protein interaction (PPI) networks to build SVM models for predicting the recurrence and prognosis of colon cancer, and both achieved an accuracy of more than 80% [26]. Finally, the DT C5.0 is more advantageous than traditional prediction methods because it is not affected by nonlinearity as well as covariance of variables. In this study, the AUC of C5.0 model was 0.929, which is the best prediction among these models.

The limitation of this study is that the results were limited by sample size, and further prospective studies with larger sample sizes are needed to validate the findings. In addition, more types of biomarkers can be collected from NSCLC patients to make the model more accurate.

In summary, the results showed that the high expression level of miR-20a and miR-197, the low expression level of miR-145 and miR-30a were strongly associated with poorer prognosis in NSCLC patients, and the DT C5.0 model may serve as a novel, accurate, non-invasive method for prognosis of NSCLC.

## Materials & methods

### Study population

The participants were from patients with primary NSCLC in the First Affiliated Hospital of Zhengzhou University, Henan Cancer Hospital and Henan Chest Hospital, from Jun. 2016 to Feb. 2017, and met the international 8th edition lung cancer stage classification criteria. The following are the inclusion criteria: (I) Patients with pathologically diagnosed primary non-small cell lung cancer; (II) Without undergone surgical resection, chemotherapy or radiation therapy; (III) Without other organ malignancies; (IV) Good compliance. The following are exclusion criteria: (I) Pregnant or lactating patients; (II) Patients with major organ function failure.

### Survey content and follow-up

The study was approved by the Ethics Committee of Zhengzhou University, and all participants were informed of the purpose of the study and voluntarily signed an informed consent form. Overall survival (OS) was defined as the period from the date of pathological diagnosis to the date of death or the follow-up cut-off time (months). The follow-up deadline was August 15, 2021. According to WHO criteria, a smoker is defined as someone who has smoked for more than 6 months cumulatively. Drinkers were defined as drinking at least 20 g of pure alcohol at least once a week.

### Main instruments and reagents

Total RNA was extracted from peripheral blood using the RNA Prep Pure Blood kit (Tiangen Biotech Co., Ltd.) according to the manufacturer protocol and quantified using NanoDrop™ 2000 (Thermo Fisher Scientific, Inc.). Total RNA (300 ng for each participant) was reverse transcribed into cDNA using the Fast-King RT kit (Tiangen Biotech Co., Ltd) according to the manufacturer's recommendations.

RT-qPCR amplification was performed using the miRcute Plus miRNA qPCR Detection kit (Tiangen Biotech Co., Ltd). The PCR reaction was performed using the 7500 Fast Real-time PCR System (Thermo Fisher Scientific, Inc.) with the following program: Initial denaturation at 95˚C for 15 min, followed by five cycles of 94˚C for 20 s, 65˚C for 30 s and 72˚C for 34 s without collecting fluorescent signals and 40 cycles of 94˚C for 20 s and 60˚C for 34 s during which fluorescent signals were collected. The dissolution curve was drawn at 60˚C for 30 s and at 95˚C for 15 s. The relative expression levels were calculated using the 22^−△△Ct^ method.

### Establishment of models

#### Data transformation

The expression of 11 miRNAs did not conform to the normal distribution, and Reciprocal transformation, logN transformation, Log10 transformation, Exponential transformation, and Square root transformation were tried. The results showed that the Log10 transformation was the most effective.

#### Data standardization

In this study, the data were standardized so that the ranged between 0 and 1.

#### Data grouping

Based on the simple random sampling of the partitioned nodes, the training and prediction sets were divided according to the ratio of 3:1, and the seed number was set as 1,211,492.

#### Data mining model establishment

Data mining models were established using five algorithms: support vector machine (SVM), artificial neural network (ANN) model, Decision tree C5.0 (DT C5.0) model, bayesian neural networks (BNN) and Fisher discriminant analysis based on SPSS Clementine 12.0 software (SPSS, Chicago).

SVM is one of the two-class classification models. Its basic definition is a linear classifier that maximizes the interval in a feature space. SVM maps the data into a high-dimensional space, using a kernel function that is typically nonlinear [27]. In this study, the kernel function was set as polynomial kernel function (Polynomial).

ANN is a simulation of a logic algorithm by imitating the information processing function of the human brain [28]. ANN is an algorithm that has self-learning capabilities. A three-layer ANN was implemented in our study.

Decision tree is a basic classification and regression method. This study uses a classification decision tree, which has a tree-shaped structure and consists of two parts: nodes and directed edges [29].After repeated training and optimization, the parameters of the decision tree C5.0 model constructed in this study.

BNN are suitable for expressing and analyzing uncertain and probabilistic events, and can make inferences from incomplete, inaccurate or impossible to judge information. Bayesian neural network is generally more accurate and robust than conventional neural networks, especially when the training data set is small [30].

Fisher discriminant analysis Fisher discriminant analysis was proposed by British statistic scientist Fisher in the 1930s. Fisher is a relatively classic method in linear learning [31]. It is widely used in classification models and is also a relatively traditional statistical method.

#### Model evaluation

This study assessed sensitivity, specificity, accuracy positive predictive value (PPV), negative predictive value (NPV), and area under the curve (AUC) to estimate the models. The Kaplan–Meier method was used to draw survival curves, the log-rank test was used to compare overall survival curves, and whether compliance with the proportional hazards (PH) assumption was evaluated, and the proportional hazards hypothesis test based on Schoenfeld residuals was used for further evaluation.

#### Cox proportional hazards regression model

The variables finally included in the Cox proportional hazards regression model in this study were: age, smoking, clinical stage, miR-16, miR-20a, miR-210, miR-145, miR-223, miR-197 and miR-30a. The stepwise backward method is used, with the default Wald test. The standard P value for variable elimination is 0.1, and the standard P value for inclusion is 0.05. Insignificant variables are eliminated to obtain the optimal model.

### Statistical analysis

**Fig. 2:**
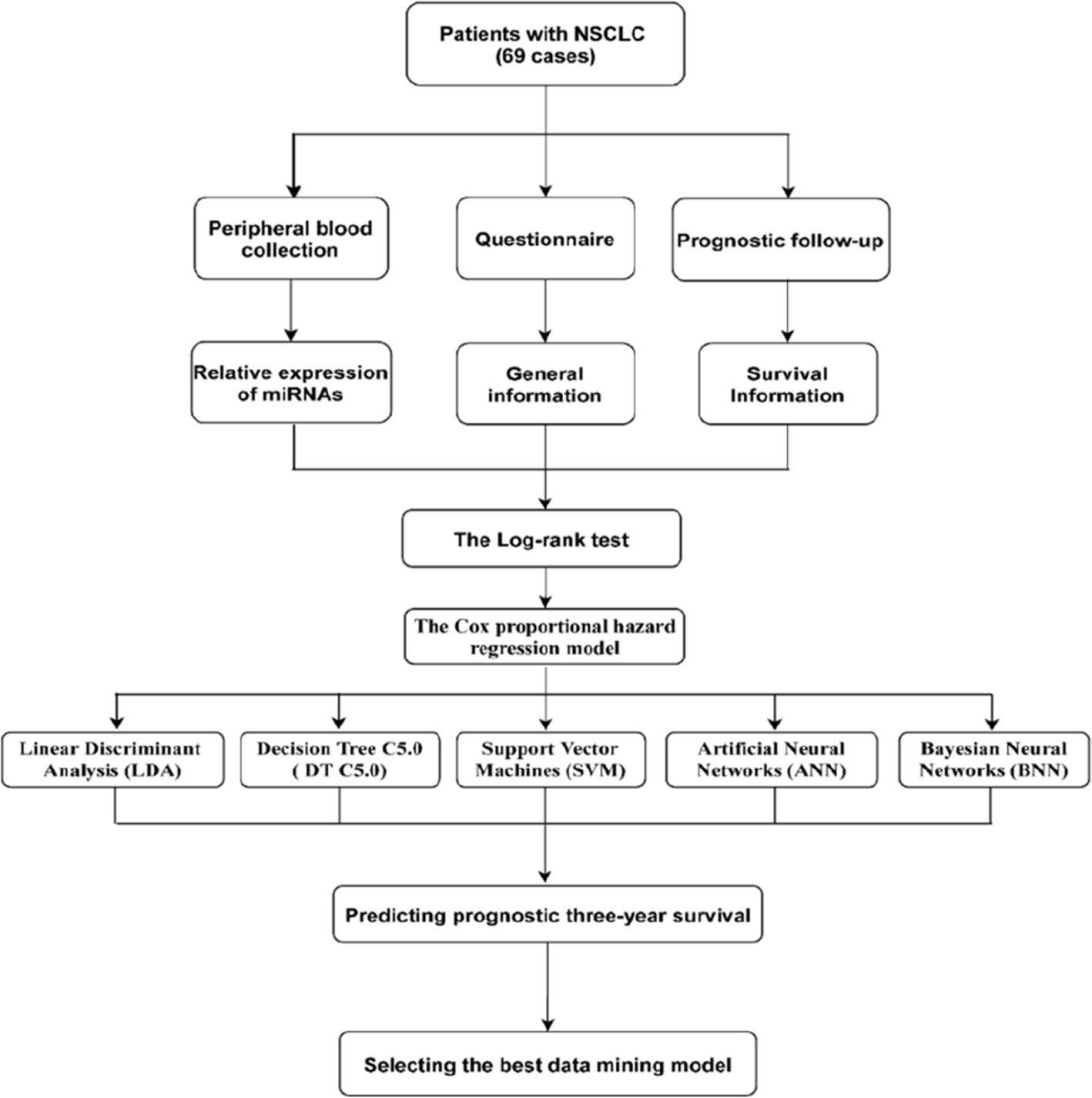
The technical roadmap for constructing a prognosis prediction model for NSCLC

The data were analyzed using SPSS 25.0. Non-normally distributed data were described by medians and quartiles. Survival times were expressed as median survival time(T_50_) and its interquartile range (T_75_, T_25_). Quantitative data were compared using the Mann–Whitney U test. The log-rank test was used for comparison of overall survival curves. The multi-factor analysis was performed by using Cox proportional hazard regression model. The significance level α was adjusted by 0.15 in order not to omit important variables and to improve the predictive power of the final model. The technical roadmap of this study was shown in Fig. 2.

### Supplementary Information

**Additional file 1.**

## Data Availability

The datasets used and analyzed during the current study are available from the corresponding author on reasonable request.
