# Supplementary material for: Prognostic value of plasma microRNAs for non-small cell lung cancer based on data mining models
Source: BMC Cancer. 2024 Jan 10;24:52. doi: 10.1186/s12885-024-11830-9 (PMC10777550; doi:10.1186/s12885-024-11830-9)
Supplement: Supplementary file 1 — Additional file 1. [file 12885_2024_11830_MOESM1_ESM.docx]

Supplementary material

Model parameter settings

Data mining model establishment

Data mining models were established using five algorithms: support vector machine (SVM), artificial neural network (ANN) model, Decision tree C5.0 (DT C5.0) model, bayesian neural networks (BNN) and Fisher discriminant analysis based on SPSS Clementine 12.0 software (SPSS, Chicago)

SVM is one of the two-class classification models. Its basic definition is a linear classifier that maximizes the interval in a feature space. The learning strategy of the support vector machine is to maximize the interval and maximize the distance from the nearest point to the hyperplane. In our reaserch, after repeating training, the SVM parameter settings were: Use partitioned data: no; Mode: Expert; Kernel type: Polynomial; Gamma: 1; Stopping criteria:1.0E-3.

Artificial neural network is a simulation of a logic algorithm by imitating the information processing function of the human brain. The parameters of the artificial neural network model in this study are set as follows: In the model tab, the name of the model: automatic; use partitioned data: checked; method: exhaustive pruning; sample: 50%; set random seed: unchecked ; Stop: time (mins) 1 min; Optimize: memory. In the Options tab, Continuously train existing models: unchecked; Use binary set encoding: checked; Show feedback graphics: checked; Model selection: Use best network; In the Expert tab, Mode: Expert; In the Analysis tab, Model Evaluation: Calculate variable importance.

Decision tree is a basic classification and regression method. This study uses a classification decision tree, which has a tree-shaped structure and consists of two parts: nodes and directed edges.

After repeated training and optimization, the parameters of the decision tree C5.0 model constructed in this study are set as follows: In the model tab, the name of the model: automatic; use partitioned data: unchecked; output type: decision tree; group symbol : Unchecked; Use boost: checked; Number of trials: 10; Cross-validation: Unchecked; Mode: Expert; Pruning severity: 75; Minimum records per child branch: 2; Use global pruning: checked; Discriminate attributes: unchecked, in the Cost tab, use misclassification loss: unchecked; in the Analysis tab, model evaluation: Calculate variable importance.

BNN are suitable for expressing and analyzing uncertain and probabilistic events, and can make inferences from incomplete, inaccurate or impossible-to-judge information. The parameter settings of the Bayesian model in this study are as follows: In the model tab, the name of the model: automatic; use partitioned data: checked; structure type: Markov blanket; include feature preprocessing step, checked; parameter learning Algorithm: Bayesian tuning of small cell counts. In the Expert tab, Mode: Expert; Independence test: Pearson chi-square; in the Analysis tab, Model evaluation: Calculate variable importance.

Fisher is a relatively classic method in linear learning. After many times of training and optimization, the Fisher discriminant analysis model in this study is set as follows: In the model tab, model name: automatic; use partitioned data: unchecked; method: input; mode: expert; prior probability: all The groups are all equal; use Covariance Matrix: Within Groups; on the Analysis tab, Model Evaluation: Calculate variable importance.
